# A graph-based gene selection method for medical diagnosis problems using a many-objective PSO algorithm

**DOI:** 10.1186/s12911-021-01696-3

**Published:** 2021-11-27

**Authors:** Saeid Azadifar, Ali Ahmadi

**Affiliations:** Faculty of Computer Engineering, K. N. Toosi University of Technology, Tehran, Iran

**Keywords:** Gene selection, Dimension reduction, Many-objective PSO, Gene clustering, High dimensional, Repair operator

## Abstract

**Background:**

Gene expression data play an important role in bioinformatics applications. Although there may be a large number of features in such data, they mainly tend to contain only a few samples. This can negatively impact the performance of data mining and machine learning algorithms. One of the most effective approaches to alleviate this problem is to use gene selection methods. The aim of gene selection is to reduce the dimensions (features) of gene expression data leading to eliminating irrelevant and redundant genes.

**Methods:**

This paper presents a hybrid gene selection method based on graph theory and a many-objective particle swarm optimization (PSO) algorithm. To this end, a filter method is first utilized to reduce the initial space of the genes. Then, the gene space is represented as a graph to apply a graph clustering method to group the genes into several clusters. Moreover, the many-objective PSO algorithm is utilized to search an optimal subset of genes according to several criteria, which include classification error, node centrality, specificity, edge centrality, and the number of selected genes. A repair operator is proposed to cover the whole space of the genes and ensure that at least one gene is selected from each cluster. This leads to an increasement in the diversity of the selected genes.

**Results:**

To evaluate the performance of the proposed method, extensive experiments are conducted based on seven datasets and two evaluation measures. In addition, three classifiers—Decision Tree (DT), Support Vector Machine (SVM), and K-Nearest Neighbors (KNN)—are utilized to compare the effectiveness of the proposed gene selection method with other state-of-the-art methods. The results of these experiments demonstrate that our proposed method not only achieves more accurate classification, but also selects fewer genes than other methods.

**Conclusion:**

This study shows that the proposed multi-objective PSO algorithm simultaneously removes irrelevant and redundant features using several different criteria. Also, the use of the clustering algorithm and the repair operator has improved the performance of the proposed method by covering the whole space of the problem.

## Background

In recent years, microarray technology has made feasible the simultaneous monitoring of thousands of genes. In particular, this type of technology has been successfully applied as a fruitful source of data in bioinformatics research in order to categorize, identify, and express thousands of genes in a wide range at the same time. Gene selection plays a crucial role in the diagnosis of various diseases. Gene expression databases tend to contain large volumes of features, but low numbers of samples. Such databases often include immense amounts of irrelevant and unnecessary attributes where only certain minute parts of genes are related to a given disease [1]. As a result, gene selection is an extremely important element in applying gene expression databases to disease diagnosis, effectively.

Gene selection methods can be classified into four categories: filter, wrapper, hybrid, and embedded [2, 3]. The filter method is related to the problems in which gene selection is carried out independently of any learning algorithm. In other words, gene selection is performed as a separate preprocessing task. A detailed and accurate statistical analysis of the selected genes is essential to carry out the gene selection method without the help of any learning model. The wrapper approach applies a defined learning algorithm in order to establish the efficiency and usefulness of the selected subsets. Wrappers are capable of producing much better results; however, they are much more costly to set up and often also involve analyzing a large number of genes. The hybrid approach combines the filter and wrapper methods and tries to exploit both of them. Finally, the embedded approach seeks to perform gene selection as a part of the learning process and is specific to a given learning sample.

A number of population-based evolutionary algorithms, including genetic algorithm (GA) [4–8], ant colony optimization (ACO) [9–12], tabu search (TS) [13, 14], simulated annealing (SA) [15, 16], and particle swarm optimization (PSO) [17–20] have attracted significant attention in the field of bioinformatics because of their ease of use and acceptable results in tackling the challenge of gene selection [21, 22]. However, most of these methods suffer from some common issues, including: (1) requiring a large amount of computational time, (2) an inability to produce acceptable outcomes because they do not focus sufficiently on reducing the size of the selected genes, and (3) the need to carry out a very large number of iterations and parameters in order to tune them. A further drawback of such population-based approaches is that they normally have only one main objective—either to increase the classification accuracy or to reduce the number of selected genes—and do not take sufficient account of other objectives, such as the strength of the relevancy between genes.

In order to tackle the above-mentioned issues, a gene selection method called MaPSOGS is proposed in this paper based on a many-objective PSO algorithm. Specifically, this paper proposes a hybrid filter-wrapper approach based on a many-objective PSO algorithm. In the proposed method, a graph clustering model is used to group the genes into several clusters. To this end, the initial set of genes are filtered using the Fisher score and then, the remaining genes are represented as a graph. Moreover, a novel operator called "repair operator" is proposed to improve the solutions in the PSO algorithm by selecting the genes from different clusters. This can make a more diverse subset of genes leading to an improvement in the performance of the classification methods. The proposed approach contains a number of contributions compared to other well-known approaches:

1. Although other approaches only consider the direct relevancy between genes, the proposed method uses the criterion of edge centrality to clearly measure the intensity of the relevancy between the genes.
2. The proposed method uses a many-objective PSO algorithm based on several important objectives for gene selection including: increasing the classification accuracy, reducing redundancy, reducing the rate of false positives, and minimizing the size of the final selected set of genes.
3. The proposed method applies a graph clustering algorithm to group similar genes together as a cluster and then uses a repair operator to ensure that the entire space of the genes is explored in the search process.
4. Determining the number of genes that should be selected is an important challenge in gene selection. This is due to the fact that the number of relevant genes is unknown; thus, the optimum number of genes to be selected is also unknown. However, in the proposed method, the optimum number of genes to be selected is measured automatically based on the overall structure of the original genes and the similarity between them.

In the literature, many studies have been conducted to develop gene selection models based on metaheuristic algorithms. A hybrid gene selection method is proposed in [20], which is based on an mRMR (minimum redundancy maximum relevance) filter. This method employs the mutual information between genes to select an optimal set of genes. In [21], a gene selection method called GANN is proposed based on the combination of genetic algorithm (GA) and a neural network model. Shreem et al. [23] proposed a gene selection method called R-m-GA, which combines the ReliefF, mRMR, and GA algorithms. To this end, a set of candidate genes is selected by applying the ReliefF algorithm. Then, the redundancy of the candidate genes is reduced by applying the mRMR algorithm. Finally, the genetic algorithm is used to select the final subset of genes using an IB1 classifier.

The particle swarm optimization (PSO) algorithm is used by many scholars to develop effective models for gene selection [24]. A feature selection method based on the univariate filter model and the PSO-based wrapper model was developed in [25]. Inbarani et al. [26] proposed a hybrid model that was adapted for medical applications. In their work, to improve disease diagnosis in medical datasets, a hybrid feature selection based on PSO and rough sets theory is applied. The PSO algorithm and the support vector machine (SVM) classifier were also integrated in [27] for feature selection and parameter optimization. Also, a distributed parallel architecture is used to overcome the high computational complexity in high dimensional datasets.

A gene selection method is introduced in [28] based on a recursive PSO algorithm. In this method, the dimensions of genes with large spaces are reduced in an iterative step. To this end, the filtering-oriented ranking approaches are applied with the recursive PSO algorithm to achieve an appropriate subset of genes. A hybrid gene selection method called IG-ISSO is proposed in [29] which is based on the combination of IG and improved simplified swarm optimization. In particular, the IG approach is applied to choose more effective genes, and then, the improved simplified swarm optimization approach is utilized to search for the optimum set of genes. In [18], the authors proposed a model to select genes based on the black hole embedding in BPSO algorithm. This model is able to increase the efficiency of the BPSO algorithm by improving both exploration and exploitation phases in the search process. In [30], the combination of the black hole algorithm and decision tree is used to propose an effective gene selection method. Chuang et al. [28] proposed a gene selection method that employs the combination of the BPSO, genetic algorithm, and KNN classifier. Sahu et al. [31] proposed a two-phases gene selection method. In the first phase, the initial genes are split into a number of clusters by using the K-MEANS algorithm. Then, the genes of each cluster are ranked using the SNR score, and the genes with the highest scores are selected as a new subset. In the second phase, the new produced subset of genes is used as the input of the PSO algorithm to obtain the final subset. Xi et al. [32] proposed a gene selection method based on the binary quantum-behaved particle swarm optimization (BQPSO) for cancer classification. In [33], a PSO algorithm called PPSO has been developed for gene selection. PPSO uses a new representation space to decrease the search space and also uses a new fitness function to better evaluate the solutions. In [34], an improved version of the PSO algorithm is introduced to improve the performance of the KNN classifier. To this end, the exploitation capability of the PSO algorithm is enhanced by determining the global optimal solution more efficiently. Banka et al. [35] proposed a feature selection method for high-dimensional data based on Hamming distance-based binary particle swarm optimization (HDBPSO). Hamming distance is used in their method as a proximity measure for updating the particle velocity during a binary PSO search process to select the requisite feature subsets. Another hybrid feature selection algorithm utilizing particle swarm optimization is proposed in [36]. This method, called HPSO-LS, selects the less correlated and salient feature subset by utilizing a new local search. Jain et al. [37], integrated the correlation feature selection with modified binary PSO algorithm for gene selection and cancer classification. This method eliminates irrelevant and redundant genes to choose a high-relevant subset. In [38], an approach to reduce dimensionality in a medical dataset was developed using PSO-based regression. Moreover, the Bayesian information criterion is combined with PSO and logistic regression as a fitness function.

There have been several studies that combine GA with PSO to benefit from both their advantages and cover their drawbacks. Li et al. [39] presented a gene selection method using a hybrid of PSO/GA and SVM as a classifier. A hybrid PSO/GA algorithm is proposed by [40] along with Artificial Neural Networks (ANNs) to uncover biomarkers from microarray data. In [41], BPSO and combat genetic algorithms (CGA) are used to reduce the number of genes in gene expression and achieve a low classification error rate.

## Methods

### Many objective optimization

Optimization problems can be divided into two categories based on the number of objective functions and optimization criteria: (1) single-objective optimization problems and (2) multi-objective optimization problems. In single-objective optimization problems, there is a target function with only one criterion in which the purpose is to find an optimal value to maximize or minimize this function. On the other hand, multi-objective optimization problems contain several objective functions with some conflicting criteria. The purpose of these problems is to find an optimal solution by considering all the conflicting criteria. Therefore, the optimization process in multi-objective problems is more critical than single-objective problems. A many-objective optimization problem is a multi-objective problem with at least four objective functions, which can be represented as follows [42]:

1 $$\begin{aligned} & minimize\; F\left( x \right) = \left( {f_{1} \left( x \right),f_{2} \left( x \right), \ldots ,f_{m} \left( x \right)} \right)^{T} , \\ & subject\, to\; x \in {\Omega } \\ \end{aligned}$$

where the decision vector $x = \left( {x_{1} ,x_{2} , \ldots , x_{n} } \right)$ is a member of the nonempty decision space ${\Omega }$, and the objective function $F$ consists of $m \left( {m \ge 4} \right)$ objective functions.

The Pareto dominance strategy is mainly applied to evaluate the solutions of the many-objective optimization problems according to the Pareto optimal solution. The definitions of the Pareto dominance and Pareto optimal solution are represented in the following [42]:

***Theorem 1: Pareto dominancePareto dominance.***

[43] Given two solutions $x , y \in {\Omega }_{f}$ and their corresponding objective vectors $F\left( x \right), F\left( y \right) \in R^{m}$, ${\varvec{x}}$ dominates ${\varvec{y}}$ (denoted as ${\varvec{x}} < {\varvec{y}}$) if and only if $\forall i \in \left\{ {1, 2, \ldots ,m} \right\}, f_{i} \left( x \right) \le f_{i} \left( y \right)$ and $\exists j \in \left\{ {1, 2, \ldots ,m} \right\}, f_{j} \left( x \right) \le f_{j} \left( y \right)$.

***Theorem 2: Pareto optimal solution.*** A solution $x^{*} \in {\Omega }_{f}$ is Pareto optimal if there is not any other solution $x \in {\Omega }_{f}$ that dominates $x^{*}$.

### Proposed method

**Fig. 1:**
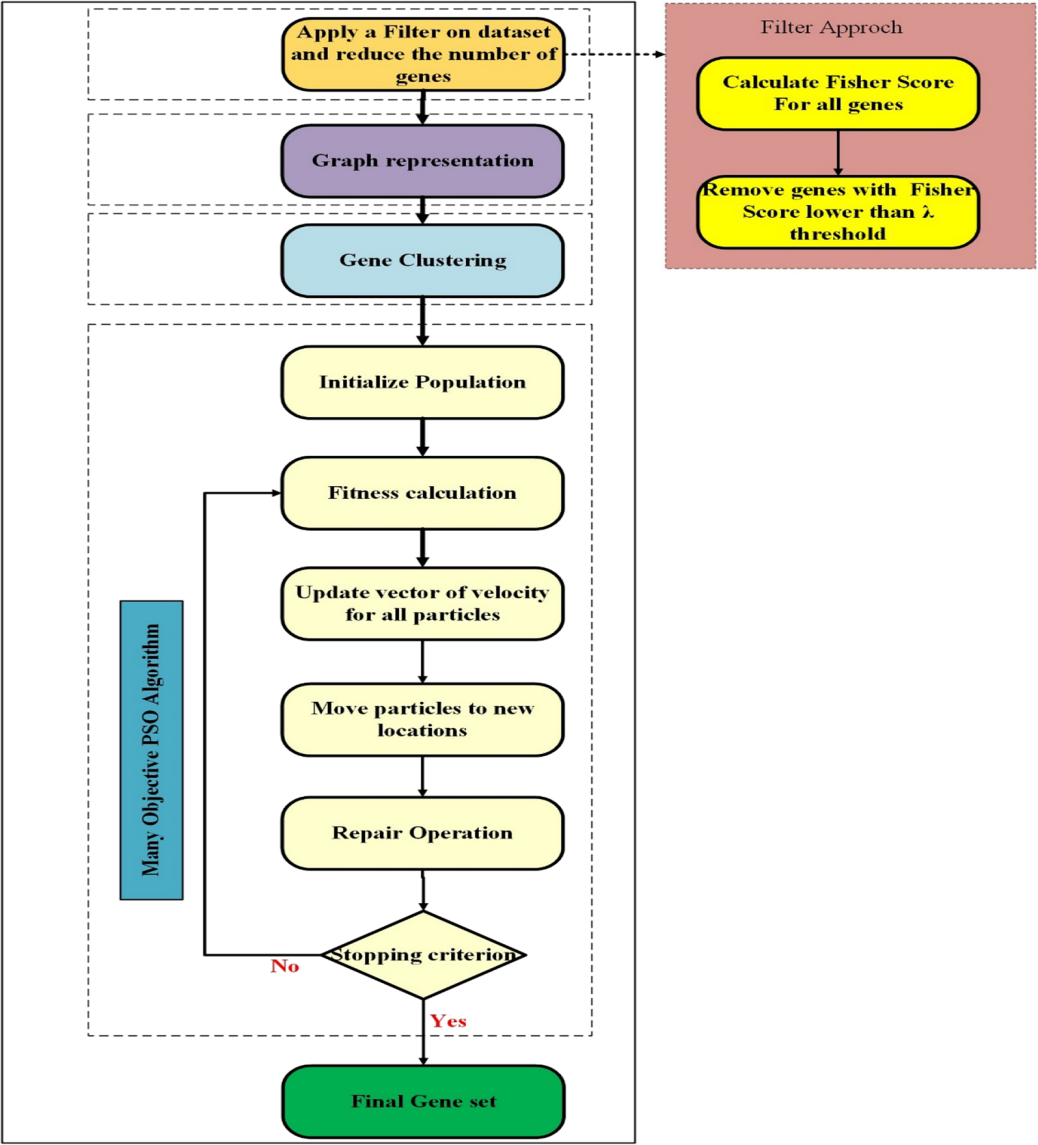
The Overview of the proposed method

In this section, we aim to introduce our proposed gene selection method for medical diagnosis problems which is called MaPSOGS. Figure 1 indicates an overview of the proposed method. This method consists of four main steps: (1) filtering out of genes, (2) graph representation, (3) genes clustering, and (4) genes selection. In the first step of the proposed method, the Fisher score is used to filter out those genes whose Fisher score values are less than a threshold value. In the second step, a graph is represented in which the reduced set of genes are the nodes and their relationships are used to construct the edges. Then, a clustering approach is applied to the graph to cluster the genes into appropriate groups. Finally, a many-objective optimization method based on the PSO algorithm is employed to select the final set of genes according to different objective functions. The details of the main steps of the proposed method are discussed in the following subsections.

### Filtering out of genes

Real-world datasets mainly contain a large number of genes, while in most cases, only a very small number of them are appropriate and other genes are irrelevant or redundant. Moreover, processing large-scale datasets with enormous genes is very time-consuming. Therefore, in this step, we aim to provide an effective mechanism to filter out irrelevant or redundant genes from the original set of genes. This helps to ease the process of selecting genes using the proposed many-objective optimization mechanism. To do this, we calculate the Fisher score for each gene using the following equation:

2 $$Score\left( {G_{i} } \right) = \frac{{\mathop \sum \nolimits_{k = 1}^{C} n_{i} (\overline{g}_{i}^{k} - \overline{g}_{i} )^{2} }}{{\mathop \sum \nolimits_{k = 1}^{C} n_{i} (\sigma_{i}^{k} )^{2} }}$$

where, $C$ is the number of classes in the dataset, $n_{i}$ is the number of samples in class $i$,$\overline{g}_{i}$ denotes the mean value of all patterns related to the gene $G_{i}$, and ${\overline{\text{g}}}_{{\text{i}}}^{{\text{k}}}$ and $\sigma_{i}^{k}$ denote mean and variance of class $k{ }$ related to the gene $G_{i}$. After calculating the Fisher score for all genes, a subset of them is selected using a threshold value named $\lambda$. In other words, those genes whose Fisher scores are lower than $\lambda$ are filtered out from the original set, and other genes are considered the selected genes. It is worth noting that the selected subset of genes is used in the next steps of the proposed method.

### Graph representation

In this step, the space of genes is represented as a graph to be used in the next step of the proposed method (i.e., genes clustering). To this end, the set of genes is mapped to a weighted graph $G = \left( {Ge, E, w_{Ge} } \right)$ in which $Ge = \left\{ {G_{1} , G_{2} , \ldots , G_{n} } \right\}$ is the set of genes, $E = \left\{ {\left( {G_{i} ,G_{j} } \right): G_{i} , G_{j} \in Ge} \right\}$ denotes the edges of the graph, and $w_{Ge} :\left( {G_{i} , G_{j} } \right) \to {\mathbb{R}}$ is a function representing the similarity values between the genes. It should be noted that in the graph representation model, the genes are considered as the nodes and the similarity values between the genes are used as the weights of edges in the graph. There are several approaches to calculate the similarity values between genes. Among these approaches, the Euclidean distance, the cosine similarity, and the Pearson correlation coefficient are the most popular functions used to calculate the similarity values between genes. In the proposed method, we use the Pearson correlation coefficient due to its efficiency in calculating the similarity value between two input vectors. The Pearson correlation coefficient between two genes $G_{i}$ and $G_{j}$ is calculated as follows:

3 $$w_{ij} = \left| {\frac{{\mathop \sum \nolimits_{p} \left( {x_{i} - \overline{{x_{i} }} } \right)\left( {x_{j} - \overline{{x_{j} }} } \right)}}{{ \sqrt {\mathop \sum \nolimits_{p} \left( {x_{i} - \overline{{x_{i} }} } \right)^{2} } \sqrt {\mathop \sum \nolimits_{p} \left( {x_{j} - \overline{{x_{j} }} } \right)^{2} } }}} \right|$$

where $x_{i}$ and $x_{j}$ represent the vectors of the genes $G_{i}$ and $G_{j}$, respectively. Moreover, $\overline{{x_{i} }}$ and $\overline{{x_{j} }}$ denote the mean values of the vectors $x_{i}$ and $x_{j}$ between $p$, respectively.

In the proposed method, the weights between the genes in the graph are normalized using a nonlinear scaling function to improve the effectiveness of the proposed method. Therefore, the weights of the graph are mapped to a new range [0, 1]. To this end, the weights of the graph (the similarity values between genes) are normalized using the following equation [44]:

4 $$\hat{w}_{ij} = \frac{1}{{1 + {\text{exp}}( - \frac{{w_{ij} - \overline{w}}}{\sigma })}}$$

where $w_{ij}$ is the similarity value between the genes $G_{i}$ and $G_{j}$, $\overline{w}$ and $\sigma$ are the mean and standard deviation of all calculated similarity values, respectively.

### Genes clustering

In this step, a graph clustering approach is used to cluster the genes into appropriate clusters. The main purpose of this step is to split up the initial genes into a number of clusters according to their similarity values. Therefore, similar genes can be assigned to the same cluster while their similarity values with the genes of other clusters will be low. In the proposed method, we use the Louvain algorithm [45] as a graph clustering approach. It should be noted that this graph clustering approach is applied to the graph which is constructed in the graph representation step. To improve the efficiency of the clustering approach, the initial graph is updated by removing the edges whose weights are less than the threshold $\theta$. The main idea of the Louvain algorithm is to perform a fast and efficient mechanism to detect the communities (clusters) by maximizing the modularity function. To this end, at first, each node is considered as a cluster, and then the clustering procedure is done in two iterative steps as follows:

**Step 1:** For each node $i$, the benefit of assigning this node to cluster C is calculated using the following equation:

5 $$\Delta Q = \left[ {\frac{{\mathop \sum \nolimits_{in} + k_{i,in} }}{2m} - \left( {\frac{{\mathop \sum \nolimits_{tot} + k_{i} }}{2m}} \right)^{2} } \right] - \left[ {\frac{{\mathop \sum \nolimits_{in} }}{2m} - \left( {\frac{{\mathop \sum \nolimits_{tot} }}{2m}} \right)^{2} - \left( {\frac{{k_{i} }}{2m}} \right)^{2} } \right]$$

where $\mathop \sum \limits_{in}$ is the total weights in cluster $C$, $\mathop \sum \limits_{tot}$ is the total weights of the edges that are connected to the nodes of cluster $C$, $k_{i}$ is the degree of node $i$, $k_{i, in}$ indicates the sum of the weights of the edges between node $i$ and other nodes in its community, and $m$ is the sum of the weights of all edges in the graph.

**Step 2:** Each node is assigned to a cluster that maximizes the modularity function. Therefore, new clusters can be obtained based on this strategy.

These two steps are repeated until the structure of clusters is no longer changed. The output of this step is a number of clusters where each cluster contains a number of similar genes. These clusters are used in the next step of the proposed method to obtain the final set of genes. The Louvain algorithm is a simple and efficient method for identifying clusters in large graphs. The computational complexity of this algorithm is $O\left( {n\log n} \right)$*,* where $n$ is the number of nodes.

### Gene selection

In this step, the final subset of genes is selected using the many-objective PSO algorithm. To do this, two important issues must be considered: (1) the representation of solutions and (2) the definition of fitness function. In the proposed method, each particle represents a solution whose length is equal to the total number of genes. If a gene is selected, the value of its position in the solution will be 1, otherwise; it will be 0. Moreover, the fitness function is defined based on several criteria that must be minimized using the optimization process.

In the many-objective PSO algorithm, first of all, the initial population of the particles is randomly generated. Then, the fitness value of each particle is calculated using the defined fitness function. It is worth noting that the fitness function is defined based on several criteria: classification error, edge centrality, node centrality, number of selected genes, and specificity metric. These criteria are considered as the objective functions that should be either maximized or minimized. Classification error evaluates the rate of the samples that are not correctly classified, which is calculated as follows:

6 $$Classification \,Error = \frac{FP + FN}{{TP + FP + TN + FN}}$$

where $FP, FN,TP, and TN$ are respectively the numbers of false positive, false negative, true positive, and true negative.

The edge centrality is a criterion for evaluating the intensity of relevancy between genes. One of the purposes of the proposed method is to select a set of genes with the least redundancy. To this end, edge centrality [46] has been applied to measure the intensity of relevancy between genes which should be minimized. The node centrality [47] is used as a criterion for assessing the influence of nodes in the graph. The higher centrality of a node leads to make a better representative for other neighboring nodes. Besides, since the true negative rate of results is highly important for medical diagnosis problems, the specificity criterion is applied to measure the ratio of correctly identified negative cases which should be maximized. The specificity metric is calculated as follows:

7 $$Specificity\left( {TNR} \right) = \frac{TN}{{TN + FP}}$$

The number of selected genes is another criterion that should be minimized. This is due to the fact that the proposed method aims to select a lower number of genes as the final subset of genes. According to the considered criteria, the many-objective fitness function for the subset of genes $GS$ is defined as follows:

8 $$minimize F\left( {GS} \right) = \left( {f_{1} \left( {GS} \right),f_{2} \left( {GS} \right),f_{3} \left( {GS} \right),f_{4} \left( {GS} \right),f_{5} \left( {GS} \right)} \right)^{T}$$

where

9 $$f_{1} \left( {GS} \right) = Classification\, Error$$

10 $$f_{2} \left( {GS} \right) = \mathop \sum \limits_{{G_{i} ,G_{j} \in G}} EC\left( {G_{i} ,G_{j} } \right); EC\left( {G_{i} ,G_{j} } \right) \,is\, the\, edge\,centrality\,between\,gene\,pair\, of\, G_{i}$$

11 $$f_{3} \left( {GS} \right) = \frac{1}{{NC\left( {GS} \right)}} ;\; NC\left( {GS} \right)\, is\, the\, sum\, of\, the\, node\, centrality\, of\, all\, genes\, in\, GS$$

12 $$f_{4} \left( {GS} \right) = \frac{1}{{Specificity\left( {TNR} \right)}}$$

13 $$f_{5} \left( {GS} \right) = \left| {GS} \right|;\; \left| {GS} \right|\, is\,the\,number\,of\, genes\, in\, GS$$

After calculating the fitness values of particles, their positions are updated based on an effective mechanism. To this end, the position of each particle is updated using its best position and also the global best position that are achieved in the previous iterations. In this regard, the position of particle $i$ is denoted by vector $x_{i}$ and also the velocity vector of this particle is defined as $v_{i}$. Then, the position of each particle $i$ is updated using the following equations [48]:

14 $$v_{i} \left( {t + 1} \right) = wv_{i} \left( t \right) + c_{1} r_{1} \left( {x_{Best,i} \left( t \right) - x_{i} \left( t \right)} \right) + c_{2} r_{2} (g_{best,i} \left( t \right) - x_{i} \left( t \right))$$

15 $$x_{i} \left( {t + 1} \right) = x_{i} \left( t \right) + v_{i} \left( t \right).t$$

where $x_{Best,i}$ and $g_{best}$ denote the best position of the particle $i$ and the best global position, respectively. Moreover, $w$ is inertia weight, $c_{1}$ and  $c_{2}$ are two positive constants, and $r_{1}$ and $r_{2}$ are two random values in the range $\left[ {0,{ }1} \right]$. It should be noted that the value of parameter $w$ is set to $w \approx \left[ {0.5,{ }0.9} \right]$ in the proposed method.

**Fig. 2:**
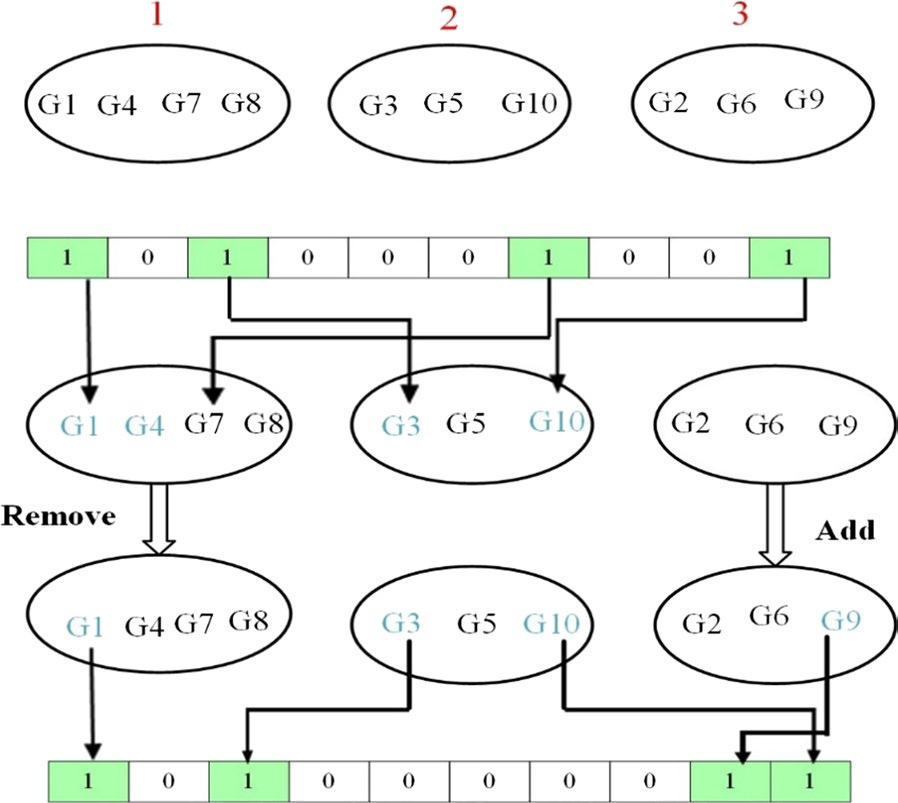
The overall schema of the proposed repair operator for a small example with ten genes. The number of clusters is set to three. The initial set of selected genes is ${\varvec{G}} = \left\{ {{\varvec{G}}_{1} ,\user2{ G}_{3} ,\user2{ G}_{7} ,\user2{ G}_{10} \user2{ }} \right\}$ that all of them belong to cluster 1 and 2 and no gene has been selected from cluster 3. After performing the repair operator, a gene with the lowest Fisher score (G7) is removed from the initial set of selected genes, and a gene from cluster 3 with the highest Fisher score (G9) is added to the final set of selected genes

In the proposed method, a repair operator is introduced which is applied to the particles in order to readjust the number of the selected genes from each cluster. To this end, we use the clusters of genes that are obtained in the previous step of the proposed method. The proposed repair operator determines that which genes should be selected or removed according to a criterion. For this purpose, at the beginning of the PSO algorithm, the effectiveness of each gene is calculated using the Fisher score (Eq. (2)). Then, the obtained scores are mapped to a new range [0, 1] to use as the probability of selection or deletion of each gene in the repair process. The main purpose of the proposed repair operator is to increase the diversification of the selected genes. To this end, it tries to select the genes from all clusters uniformly, instead of selecting the genes from a small number of clusters. Therefore, if the number of selected genes from a cluster is less than $\omega$, a number of genes with the lowest Fisher score in the selected subset will be replaced with the genes in this cluster with the highest fisher score. The main advantage of the proposed repair operator is to enhance the diversity of selected genes leading to an improvement in the performance of the proposed gene selection method. Figure 2 illustrates the overall schema of the proposed repair operator.

The proposed many-objective PSO algorithm performs iteratively until the stopping criteria are satisfied. The stopping criteria are satisfied if the maximum number of iterations is reached or there is no change in the positions of particles. Finally, the best particle obtained by the many-objective PSO algorithm is considered as the final solution, which contains the genes selected as the output of the proposed gene selection method. The pseudo-code of the proposed gene selection method is represented in Algorithm 1.
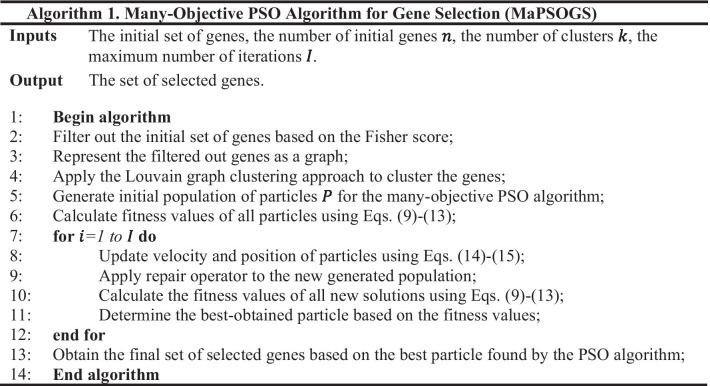


### Computational complexity analysis

In this section, the computational complexity of the proposed method is calculated. In the first step, the relevance values of the genes are evaluated using the Fisher measure. The computational complexity of this step is $O\left( {ncp} \right)$ where $n{ }$ is the number of genes in the original set, $c$ is the number of classes, and $p$ denotes the number of samples. The second step of the proposed method represents the gene space as a graph which its complexity is $O\left( {m^{2} p} \right)$ where $m$ is the number of the genes after applying the Filter method. In the third step, the Louvain community detection algorithm is used to cluster the genes into several clusters which is performed with the complexity of $O\left( {m\log m} \right)$. Then, the proposed many-objective PSO algorithm is used to select the final set of genes. In this step, the fitness value of each particle should be calculated. The computational complexity of the SVM classification algorithm is $O\left( {p^{2} .S} \right){ }$ where $S = \omega {*}k$. Moreover, the computational complexity of calculating the edge centrality and node centrality is $O\left( {m^{2} } \right)$. Therefore, the calculation of the fitness function has a computational complexity $O\left( {I.A.p^{2} .S + I.A.m^{2} } \right)$ where $I$ represents the maximum number of iterations and $A$ is the number of particles. Also, in each iteration of the algorithm, it is necessary to apply the repair operator on all particles. The complexity of the repair operator is ${ }O\left( {I.A.k.m} \right)$, where $k$ represents the number of clusters. Therefore, the computational complexity of this step of the proposed method is ${ }O\left( {I.A.k.m + I.A.p^{2} .S + I.A.m^{2} } \right)$, which can be reduced to ${ }O\left( {m^{2} + P^{2} .S} \right)$. According to the computational complexities of all steps, the final computational complexity of the proposed method will be ${ }O\left( {n.c.p + m^{2} .p + m.\log m + m^{2} + p^{2} .S} \right)$ which is reduced to $O\left( {m^{2} .p + p^{2} .S} \right)$.

## Results

To evaluate the performance of the proposed gene selection method, several experiments are performed, and their results are discussed in this section. These experiments are conducted on seven gene expression datasets that are gathered from http://www.biolab.si/supp/bi-cancer/projections/info/leukemia.html. The characteristics of these datasets are shown in Table 1. Moreover, the accuracy of classification methods is measured based on the selected set of genes and also the number of selected genes. In other words, the higher accuracy value and the lower number of selected genes show the higher performance for the gene selection models. To this end, different classification methods are employed, including Support Vector Machine (SVM), Decision Tree (DT), and K-Nearest Neighbors (KNN). Furthermore, the Weka software [49] is applied to build classifiers in the experiments. The proposed method is compared with several state-of-the-art gene selection models that are briefly described in the following:

- **Geometric particle swarm optimization (GPSO) **[50]: a gene selection method based on the PSO algorithm that applies binary representation in Heming space.
- **PSO**: a gene selection method which obtains a subset of genes using the basic version of the PSO algorithm and evaluates the selected genes by means of an SVM classifier.
- **RMA **[51]: a gene selection method based on the recursive Memetic Algorithm (MA). The recursive strategy of this algorithm is used to make a balance between the exploitation and exploration phases of the search process.
- **RPSW **[52]: a gene selection method based on the combination of the Return PSO Algorithm and different filtering ranking methods. It also applies a linear SVM weight vector to select the primary gene subset.
- **IG-ISSO **[29]: a hybrid method which uses information gain (IG) as the filtering method to select a subset of genes that is most informative based on the amount of IG. Moreover, it uses an improved simplified swarm optimization (ISSO) algorithm to perform the search process.
- **Hybrid BPSO-BBHA **[21]: a gene selection method which incorporates the Binary Black Hole Algorithm (BBHA) in the BPSO algorithm to facilitate and improve the efficiency of the BPSO.
- **PSOC4.5 **[53]: a gene selection method based on the PSO algorithm and decision tree classifier. It uses the search capabilities of the PSO algorithm to interpret the knowledge of C4.5.

**Table 1:** The characteristics of the used datasets

Dataset	No. of genes	No. of classes	No. of samples
AMLGSE2191	12,616	2	54
Colon	7464	2	36
DLBCL	7070	2	77
Leukaemia	5147	2	72
Prostate	12,533	2	102
MLL	12,533	3	72
SRBCT	2308	4	83

### Performance comparison

In this subsection, the experimental results are reported in terms of the number of selected genes and the average classification accuracy over ten independent runs for different gene selection methods. It is worth mentioning that a training set is considered to pick the final subset of genes. Also, a test set is applied to evaluate the selected genes. Table 2 shows the performance of different gene selection methods based on the number of genes which are selected by these methods. As we can see from this table, the proposed method achieves better results than the other models in most cases for the used datasets. The average number of selected genes for the proposed method is the best value among all the compared methods. Therefore, it can be concluded that the proposed gene selection method can significantly reduce the initial space of the genes by selecting the lower number of them than the other compared models. The average number of selected genes for the proposed method is 5.14 while the second-best value is obtained by the RMA model which is equal to 5.42.

**Table 2:** Performance comparison of different gene selection methods based on the number of selected genes

Dataset	Gene selection method							
	MaPSOGS	Geometric PSO	PSO	RMA	RPSW	EPSO	Hybrid BPSO-BBHA	PSOC4.5
AMLGSE2191	10	32	98	12	46	28	37	34
Colon	3	19	81	2	37	23	16	12
DLBCL	4	21	88	4	28	16	12	8
Leukaemia	6	18	85	5	24	21	28	24
Prostate	4	18	99	5	16	14	10	11
MLL	3	9	94	4	18	19	8	12
SRBCT	6	12	78	6	16	31	18	10
Average	5.14	18.42	89	5.42	26.42	21.71	18.42	15.85

The performance of the compared gene selection methods is also evaluated based on the accuracy of classification models which are applied to the selected subset of genes. To this end, the leave one out cross validation approach is used to measure the accuracy of classification models. In these experiments, we use three different classification models including SVM, DT, and KNN classifiers. The results of experiments are reported for SVM, DT, and KNN classifiers in Tables 3, 4, and 5, respectively. As it can be seen from these results, the proposed method often has higher classification accuracy than the other methods in most cases. In addition, the standard deviation value and the rank of each method are reported in these tables. The results demonstrate that the proposed method achieves the first rank in comparison to other gene selection models based on all used datasets and all considered classifiers. Table 3 shows that in all cases except for the Colon dataset, the proposed method has the best performance among all the other gene selection methods. On the other hand, while the RMA approach has the best performance for the Colon dataset, the proposed method takes the second ranking position.

**Table 3:** The results of different gene selection methods over different datasets based on SVM classifier

Dataset		Gene selection method							
		MaPSOGS	Geometric PSO	PSO	RMA	RPSW	EPSO	Hybrid BPSO-BBHA	PSOC4.5
AMLGSE2191	Acc (%)	**100 (1)**	89.01 (7)	82.29 (8)	96.29 (3)	90.48 (6)	91.63 (5)	94.26 (4)	96.88 (2)
	Std	1.58	2.68	3.54	1.80	2.11	2.37	3.01	2.93
Colon	Acc (%)	99.52 (2)	89.93 (7)	86.63 (8)	**100 (1)**	91.38 (5)	91.01 (6)	92.25 (4)	95.27 (3)
	Std	1.90	2.25	3.18	2.81	3.18	3.20	1.31	6.38
DLBCL	Acc (%)	**98.84 (1)**	86.08 (7)	83.54 (8)	95.15 (2)	94.24 (3)	91.75 (6)	92.06 (5)	92.65 (4)
	Std	1.36	0.63	0.81	0.88	2.32	1.20	0.77	1.65
Leukaemia	Acc (%)	**98.71 (1)**	86.13 (7)	82.39 (8)	96.81 (2)	91.90 (4)	90.96 (5)	89.92 (6)	93.46 (3)
	Std	1.90	1.44	1.85	1.44	1.71	1.58	0.76	1.39
Prostate	Acc (%)	**98.38 (1)**	85.96 (7)	84.55 (8)	97.41 (2)	90.51 (4)	89.14 (5)	87.92 (6)	91.81 (3)
	Std	0.33	2.10	1.60	1.29	1.21	1.60	2.76	4.51
MLL	Acc (%)	**96.38 (1)**	81.26 (8)	81.67 (7)	91.23 (3)	86.53 (5)	85.98 (6)	86.75 (4)	91.63 (2)
	Std	1.51	3.46	4.26	3.63	3.40	3.64	4.65	3.32
SRBCT	Acc (%)	**98.91 (1)**	84.56 (7)	83.28 (8)	95.37 (2)	91.50 (5)	90.11 (6)	91.70 (4)	93.03 (3)
	Std	2.42	4.44	2.50	4.87	4.80	1.49	3.63	2.98
Average	Acc (%)	**98.68 (1.14)**	86.13 (7.14)	83.48 (7.85)	96.04 (2.14)	90.93 (4.57)	90.08 (5.57)	90.69 (4.71)	93.53 (2.85)
	Std	1.57	2.43	2.53	2.39	2.67	2.15	2.41	3.31

The best results are shown in bold

**Table 4:** The results of different gene selection methods over different datasets based on DT classifier

Dataset		Gene selection method							
		MaPSOGS	Geometric PSO	PSO	RMA	RPSW	EPSO	Hybrid BPSO-BBHA	PSOC4.5
AMLGSE2191	Acc (%)	**97.08 (1)**	86.65 (7)	80.90 (8)	95.11 (2)	91.18 (5)	90.01 (6)	91.63 (4)	94.05 (3)
	Std	5.08	4.45	6.16	3.96	4.39	3.88	4.24	2.77
Colon	Acc (%)	**96.52 (1)**	87.05 (7)	84.09 (8)	94.89 (2)	91.55 (4)	89.14 (6)	90.95 (5)	92.64 (3)
	Std	2.29	3.65	4.02	2.07	4.78	3.95	2.47	3.95
DLBCL	Acc (%)	**94.57 (1)**	85.62 (7)	81.33 (8)	92.63 (2)	91.07 (3)	90.22 (5)	89.96 (6)	90.91 (4)
	Std	1.54	5.72	6.16	2.85	4.66	3.39	7.05	4.48
Leukaemia	Acc (%)	**95.42 (1)**	83.39 (7)	82.12 (8)	92.89 (2)	89.69 (4)	88.43 (6)	88.53 (5)	91.57 (3)
	Std	0.86	4.17	4.55	1.01	2.33	3.97	3.91	1.93
Prostate	Acc (%)	**96.71 (1)**	81.93 (8)	83.05 (7)	95.09 (2)	90.28 (4)	86.14 (6)	87.19 (5)	91.18 (3)
	Std	3.75	4.99	7.87	2.05	5.66	2.84	5.41	2.97
MLL	Acc (%)	**93.44 (1)**	80.98 (7)	79.24 (8)	90.93 (2)	85.87 (5)	89.88 (4)	85.28 (6)	90.22 (3)
	Std	2.12	5.83	5.75	4.16	2.49	4.18	6.17	3.63
SRBCT	Acc (%)	**95.85 (1)**	81.66 (8)	80.88 (7)	92.16 (2)	90.02 (5)	90.75 (4)	89.97 (6)	91.76 (3)
	Std	4.09	7.02	6.58	3.90	5.54	5.40	4.11	3.75
Average	Acc (%)	**95.65 (1)**	83.89 (7.28)	81.66 (7.71)	93.38 (2)	89.95 (4.28)	89.22 (5.28)	89.07 (5.28)	91.76 (3.14)
	Std	2.82	5.12	5.87	2.86	4.26	3.94	4.76	3.35

The best results are shown in bold

**Table 5:** The results of different gene selection methods over different datasets based on KNN classifier

Dataset		Gene Selection Method							
		MaPSOGS	Geometric PSO	PSO	RMA	RPSW	EPSO	Hybrid BPSO-BBHA	PSOC4.5
AMLGSE2191	Acc (%)	**95.36 (1**)	87.08 (7)	81.16 (8)	95.11 (2)	90.79 (6)	91.22 (4)	91.05 (5)	93.39 (3)
	Std	2.33	7.15	5.78	1.23	8.04	3.99	4.57	3.50
Colon	Acc (%)	**96.18 (1)**	86.76 (7)	84.88 (8)	94.10 (2)	90.55 (4)	88.21 (6)	90.36 (5)	91.45 (3)
	Std	0.24	4.77	6.42	1.70	5.49	4.87	5.12	3.10
DLBCL	Acc (%)	**93.89 (1)**	86.96 (7)	82.83 (8)	91.49 (2)	91.18 (3)	89.64 (5)	88.07 (6)	90.39 (4)
	Std	0.59	5.11	4.96	1.94	3.07	2.79	2.94	2.28
Leukaemia	Acc (%)	**93.81 (1)**	84.14 (7)	82.66 (8)	91.95 (3)	88.71 (5)	87.19 (6)	89.06 (4)	91.88 (2)
	Std	4.17	4.17	3.69	2.91	4.17	3.60	4.02	3.62
Prostate	Acc (%)	**94.25 (1)**	80.83 (8)	82.69 (7)	93.77 (2)	90.26 (3)	85.55 (6)	86.37 (5)	90.04 (4)
	Std	2.41	5.53	7.01	4.84	4.26	5.03	2.50	3.09
MLL	Acc (%)	**93.74 (1)**	81.49 (7)	80.90 (8)	91.55 (2)	83.72 (6)	88.78 (4)	86.44 (5)	90.61 (3)
	Std	1.01	3.13	4.25	3.05	3.83	4.45	3.96	3.77
SRBCT	Acc (%)	**94.90 (1)**	81.96 (7)	81.13 (8)	92.53 (2)	90.11 (4)	90.01 (5)	88.83 (6)	91.39 (3)
	Std	2.85	4.18	3.18	3.21	5.73	3.97	2.98	2.19
Average	Acc (%)	**94.59 (1)**	84.18 (7.14)	82.32 (7.85)	92.93 (2.14)	89.33 (4.42)	88.66 (5.14)	88.60 (5.14)	91.31 (3.14)
	Std	1.94	4.86	5.04	2.70	4.94	4.1	3.73	3.08

The best results are shown in bold

We report the classification accuracy for SVM, DT, and KNN classifiers in Tables 3, 4, and 5. As you can see from these numerical results, the proposed method is superior to other swarm intelligence-based gene selection methods according to all datasets. For example, for the MLL dataset, MaPSOGS obtains a 96.38% classification accuracy while for Geometric PSO, PSO, RMA, RPSW, EPSO, Hybrid BPSO-BBHA, and PSOC4.5, this value was reported 81.26%, 81.67%, 87.19%, 91.23%, 86.53%, 85.98%, 86.75%, and 91.63%, respectively. In addition, the results of these tables show that the proposed method has an average ACC of 98.68%, 95.65%, and 94.59% for the SVM, DT, and KNN classifiers, respectively. Compared to the second-ranked RMA method which has the average ACC of 96.04%, 93.38%, and 94.59%, these ACCs demonstrate improvements of 2.64%, 2.27%, and 1.66% for the proposed method. Also, the results of Table 2 indicate that the MaPSOGS method selects fewer genes than other methods. The average number of genes selected by the proposed method is 5.14.

### Sensitivity analysis of the parameters

**Fig. 3:**
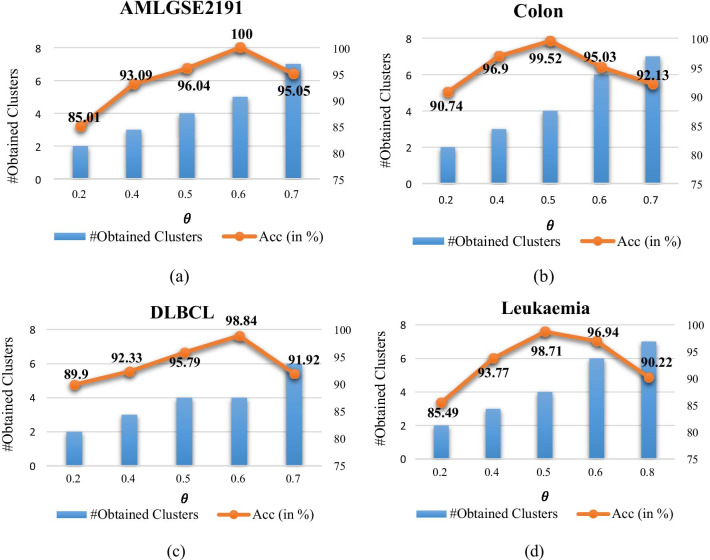
Average classification accuracy and the number of obtained clusters for different values of the parameter ${\varvec{\theta}}$ based on SVM classifier: **a** AMLGSE2191 dataset, **b** Colon dataset, **c** DLBCL dataset, and **d** Leukemia dataset

In the second step (genes clustering) of the proposed method, the edges with associated weights lower than the parameter $\theta$ will be removed from the graph. The performance of the genes clustering algorithm depends on the value of the parameter $\theta$. The value of this parameter can be set to any value in the range [0,1]. If $\theta$ is set to a small value, more edges will be considered in the graph clustering algorithm and the number of obtained clusters will be declined. On the other hand, if $\theta$ is set to a high value, the graph clustering algorithm identifies a greater number of clusters. In this section, a series of experiments are conducted to analyze the effect of parameter $\theta$ on the performance of the genes clustering algorithm. Figure 3 shows the accuracy of the proposed method based on SVM classifier in terms of different values of the parameter $\theta$ for AMLGSE2191, Colon, DLBCL, and Leukemia datasets. Moreover, the number of obtained clusters is also reported in Fig. 3. These results indicate that the higher value of the parameter $\theta$ leads to an increasement in the number of obtained clusters. On the other hand, the accuracy of the proposed method is declined when the value of the parameter $\theta$ exceeds a specific value. For instance, when the value of the parameter $\theta$ exceeds 0.6, the accuracy of the proposed method will be reduced for the AMLGSE2191 dataset. It can be concluded from these results that when the parameter $\theta$ is set to a small value, the graph clustering algorithm identifies lower number of clusters. As a result, in this case, the proposed method selects the smaller number of genes and thus, most representative genes cannot be selected to reduce the classifier accuracy.

**Fig. 4:**
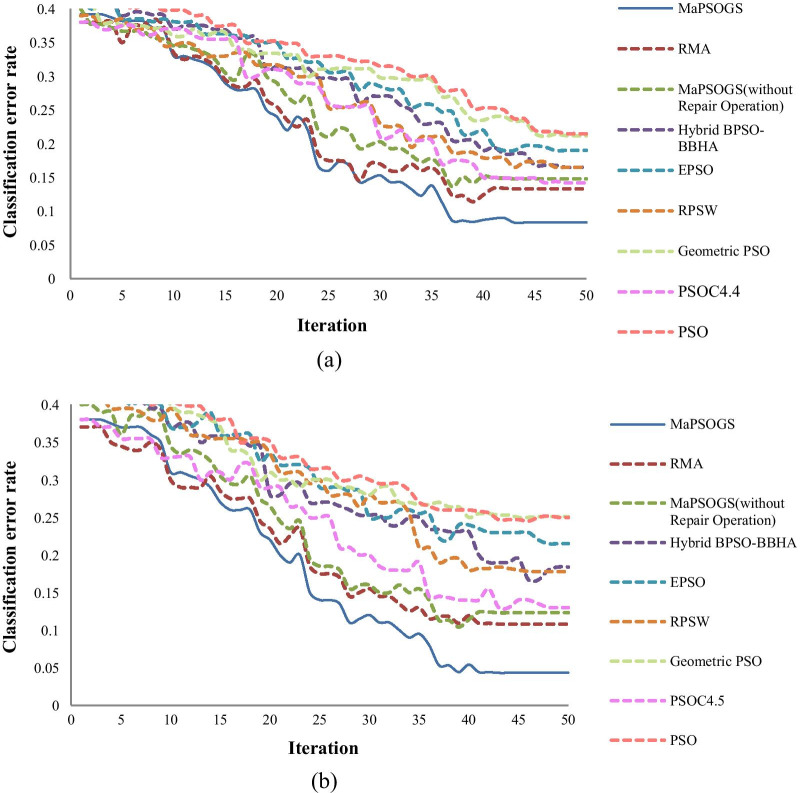
Comparison of convergence speed of MaPSOGS, RMA, MaPSOGS (without repair operator), Hybrid BPSO-BBHA, EPSO, RPSW, Geometric PSO, PSOC4.5 and PSO models based on: **a** the Colon dataset and **b** the DLBCL dataset

To investigate the convergence speed of the proposed method, the convergences of MaPSOGS, RMA, BPSO-BBHA, EPSO, RPSW, Geometric PSO, PSOC4.5, and PSO methods as well as MaPSOGS algorithm without repair operator are compared based on Colon and DLBCL datasets. Figure 4 shows the convergence speed of these methods in terms of different iterations. Figure 4 (a) shows the convergence speed for the Colon dataset. As it can be seen from this figure, the convergence speed of the proposed method is faster than other ten models. Moreover, the classification error of the proposed method is lower than the other models in most cases. The superiority of the proposed method in respect to the model without the repair operator demonstrates that the repair operator has a positive effect on the accuracy of classification models. Figure 4 (b) shows the convergence speed of the compared methods on the DLBCL dataset. These results also show that the proposed method has less classification error and a faster convergence speed in comparison to other models.

### Statistical analysis of the proposed method

In order to have a statistical analysis on the results of the proposed method and other compared methods, the Friedman test [54] is conducted. This statistical test is a nonparametric test which is used to compare the performance of different methods based on different medical datasets. In this way, the rank of each gene selection model can be obtained according to the used datasets. To this end, we use the SPSS statistics acquired by IBM [55]. In the Friedman test, hypothesis $\mathrm{H}0$ is based on the sameness of the average ranks among the groups. Rejection of the null hypothesis means that at least two groups have significant differences. In the analysis of the Friedman test results, it is impossible to determine that if the level of significance is less than the level of error, the difference between at least a pair of specimens is deducted. Since the test error is considered 5%, the level of significance must be lower than 0.05 to satisfy the constraint. Tables 6 and 7 report the Friedman test results for the proposed method compared with the other gene selection methods. In particular, Table 6Table shows the average rankings of all compared models based on different classification methods. As we can see from these results, the proposed method has the best ranking among all compared models. Therefore, it can be concluded that the proposed method is the best performer according to all considered classifiers. Table 7 shows that the Friedman test has reported a *P* value of 0.0018 for the classification accuracy of the SVM classifier. Since this is below 0.05, we can claim that the results of the proposed method are significantly different from those of other methods. Other *P* values achieved by the Friedman test for DT and KNN classifiers confirm the claim that the proposed method is significantly different from other compared methods.

**Table 6:** Average ranks of different methods on SVM, DT, and KNN classifiers

Classifier	Methods							
	MaPSOGS	Geometric PSO	PSO	RMA	RPSW	EPSO	Hybrid BPSO-BBHA	PSOC4.5
SVM	1.14	7.14	7.85	2.14	4.57	5.57	4.71	2.85
DT	1	7.28	7.71	2	4.28	5.28	5.28	3.14
KNN	1	7.14	7.85	2.14	4.42	5.14	5.14	3.14

**Table 7:** The results of the statistical test

	Classifier		
	SVM	DT	KNN
Chi-square	16.650	15.620	10.950
df	3	3	3
Asymp.Sig	0.001834	0.001369	0.011996

## Discussion

This section briefly explains why the performance of the proposed MaPSOGS method is better than other compared gene selection methods. The superiority of the proposed method is due to three main contributions that are discussed in the following.

1. Irrelevant and redundant genes severely affect the accuracy of the learning algorithms [56–58]. Thus, gene selection methods should be able to identify and remove these irrelevant and redundant genes in the original space. Most of the existing gene selection methods can effectively remove the irrelevant genes but fail to handle the redundant genes. In the univariate methods (i.e., L-Score, F-Score, and RelifF), the relevance of a gene with others is individually measured and the possible dependency between the genes will be ignored in the gene selection process. Thus, these methods cannot be able to remove the redundant genes precisely. On the other hand, most of the multivariate gene selection methods only eliminate the redundant genes without paying attention to the irrelevant genes. In this paper, we develop a novel gene selection method which can efficiently deal with both irrelevant and redundant genes. The proposed method selects the genes with minimum similarity with those of the previous selected ones using the edge centrality measure while maximizes the dependency with the target class using the error classification rate. By applying these two objective functions, the redundant and irrelevant genes have a lower probability of being selected.
2. One of the main shortcomings of existing gene selection methods is to choose the genes independently and consider the direct similarity between genes. To address this issue, the proposed method considers the intensity of relevancy between genes using the edge centrality criterion. Also, the specificity criterion is used in the proposed method to identify the negative samples leading to an improvement in the accuracy of classification models.
3. The proposed method uses a graph clustering model to cluster the genes into appropriate clusters. This leads to group the similar genes into the same cluster in which the members of each cluster are as far as possible to other clusters. Then, a repair operator is proposed to guarantee that the selected genes belong to different clusters. This operator could significantly increase the diversity of the selected genes leading to enhance the efficiency of the proposed gene selection method. Also, the repair operator increases the exploration capability of the proposed method and thus, improves the ability of the proposed method in finding the global optimal subset of the genes.
4. In order to select the optimal number of genes for the medical diagnosis, a reliable gene selection method should identify the optimal number of genes. When there are too many genes selected in a gene selection method, it increases the likelihood of selecting redundant and irrelevant genes, resulting in a reduced prediction accuracy. In contrast, too few genes may not be able to represent all of the original information. In this study, a many-objective fitness function is proposed, taking into account five main objectives, including the relevance of the gene, the redundancy of the gene, the classification error, specificity and the size of the gene subset. Therefore, redundant and irrelevant genes are selected with a low probability. Also, the selected genes provide sufficient information for diagnosis and prediction tasks. However, it is necessary to specify the number of genes in advance. The number of useful genes is hard to estimate before gene selection in actual medical applications. Whilst, the proposed method automatically determines the number of final gene set.
5. Swarm intelligence methods require consideration of the conflicting goals of exploring the search space and exploiting the best solutions. Exploration encompasses the process of generating various solutions so as to gain a global perspective on the search space. On the other hand, exploitation involves concentrating the search in a good region. A good balance between these two objectives will improve the performance of the searching method. By employing MaPSOGS, we aim to show that there is a powerful gene selection method that can balance exploitation and exploration capabilities while achieving faster convergence speed. We developed two strategies to balance between initial population generation and mutation operators in this paper. Accordingly, the node-centrality is used to enhance the exploitation in the proposed method. On the other hand, repair operator is considered as a diversification operator to avoid being stuck in local optimal by encouraging search exploration.

## Conclusion

An effective gene selection method is proposed in this paper which is based on a many-objective PSO algorithm, the strategies of the graph theory, and also graph clustering. To this end, a filter approach is used to reduce the number of genes in the original set and then, a graph clustering algorithm is applied to the graph representing the genes and their relationships to cluster the genes into appropriate clusters. Moreover, a many-objective PSO algorithm is used to obtain an optimal subset of genes according to an SVM classifier as an evaluator. To define the fitness function of the many-objective PSO algorithm, different criteria are considered including classification error, node centrality, specificity, edge centrality, and also the number of genes selected by the proposed method. In addition, a repair operator is proposed to increase the diversity of the selected genes leading to enhance the performance of the proposed method. The proposed method is compared with seven well-known gene selection methods based on different medical datasets. The experimental results demonstrated that the proposed gene selection method can significantly outperform other compared models. Several user-specified parameters are used in the proposed method which their appropriate values should be determined initially. Gene selection algorithms rely on these parameters because they control how the learning model behaves and have a considerable impact on the final prediction. In order to select the best parameter values, repeating the process of setting parameters, generating several predictions with different combinations of values, and then testing the results to pick the most accurate ones is necessary. Therefore, it is necessary to optimize the parameters to obtain the best results. Exhaustive search algorithms can be used to optimize parameters’ values. In future work, an optimization method can be used to adjust the parameters. Also, social network analysis techniques such as popularity and community detection can be used to select the final gene set.

## Data Availability

The datasets used during the current study are freely available at http://www.biolab.si/supp/bi-cancer/projections/info/leukemia.html.

## References

[1] S. Vanjimalar, D. Ramyachitra, P. Manikandan. A review on feature selection techniques for gene expression data. In: *2018 IEEE International Conference on Computational Intelligence and Computing Research (ICCIC)*. 2018. p. 1–4.

[2] Saeys Y, Inza I, Larranaga P (2007). A review of feature selection techniques in bioinformatics. Bioinformatics (Oxford, England).

[3] George V, Velanganny C (2011). Review on feature selection techniques and the impact of Svm for cancer classification using gene expression profile. Int J Comput Sci Eng Surv.

[4] Zhu Z, Ong Y-S, Dash M (2007). Markov blanket-embedded genetic algorithm for gene selection. Pattern Recognit.

[5] Li S, Wu X, Hu X (2008). Gene selection using genetic algorithm and support vectors machines. Soft Comput.

[6] Bonilla Huerta E, Duval B, Hao J-K (2010). A hybrid LDA and genetic algorithm for gene selection and classification of microarray data. Neurocomputing.

[7] Adie EA, Adams RR, Evans KL, Porteous DJ, Pickard BS (2005). Speeding disease gene discovery by sequence based candidate prioritization. BMC Bioinform.

[8] Rostami M, Berahmand K, Forouzandeh S (2021). A novel community detection based genetic algorithm for feature selection. J Big Data.

[9] Yu H, Gu G, Liu H, Shen J, Zhao J (2009). A modified ant colony optimization algorithm for tumor marker gene selection. Genomics Proteomics Bioinform.

[10] Tabakhi S, Najafi A, Ranjbar R, Moradi P (2015). Gene selection for microarray data classification using a novel ant colony optimization. Neurocomputing.

[11] Vafaee Sharbaf F, Mosafer S, Moattar MH (2016). A hybrid gene selection approach for microarray data classification using cellular learning automata and ant colony optimization. Genomics.

[12] Sun L, Kong X, Xu J, Xue Z, Zhai R, Zhang S (2019). A hybrid gene selection method based on reliefF and ant colony optimization algorithm for tumor classification. Sci Rep.

[13] Zhang H, Sun G (2002). Feature selection using tabu search method. Pattern Recognit.

[14] Shen Q, Shi W-M, Kong W (2008). Hybrid particle swarm optimization and tabu search approach for selecting genes for tumor classification using gene expression data. Comput Biol Chem.

[15] Filippone M, Masulli F, Rovetta S (2011). Simulated annealing for supervised gene selection. Soft Comput.

[16] M. Filippone, F. Masulli, S. Rovetta. Supervised classification and gene selection using simulated annealing. In: The 2006 IEEE International Joint Conference on Neural Network Proceedings. 2006. p. 3566–3571.

[17] Mohamad MS, Omatu S, Deris S, Yoshioka M (2009). Particle swarm optimization for gene selection in classifying cancer classes. Artif Life Robot.

[18] Han F, Tang D, Sun Y-W-T, Cheng Z, Jiang J, Li Q-W (2019). A hybrid gene selection method based on gene scoring strategy and improved particle swarm optimization (in Eng). BMC Bioinform.

[19] Han F (2017). A gene selection method for microarray data based on binary PSO encoding gene-to-class sensitivity information. IEEE/ACM Trans Comput Biol Bioinform.

[20] Rostami M, Forouzandeh S, Berahmand K, Soltani M (2020). Integration of multi-objective PSO based feature selection and node centrality for medical datasets. Genomics.

[21] Pashaei E, Pashaei E, Aydin N (2019). Gene selection using hybrid binary black hole algorithm and modified binary particle swarm optimization. Genomics.

[22] Han F, Tang D, Sun Y-W-T, Cheng Z, Jiang J, Li Q-W (2019). A hybrid gene selection method based on gene scoring strategy and improved particle swarm optimization. BMC Bioinform.

[23] Shreem S, Sheikh Abdullah S, Nazri MZA, Alzaqebah M (2012). Hybridizing relief, mRMR filters and GA wrapper approaches for gene selection. J Theor Appl Inf Technol.

[24] Rostami M, Berahmand K, Nasiri E, Forouzandeh S (2021). Review of swarm intelligence-based feature selection methods. Eng Appl Artif Intell.

[25] Unler A, Murat A, Chinnam RB (2011). mr2PSO: a maximum relevance minimum redundancy feature selection method based on swarm intelligence for support vector machine classification. Inf Sci.

[26] Inbarani HH, Azar AT, Jothi G (2014). Supervised hybrid feature selection based on PSO and rough sets for medical diagnosis (in Eng). Comput Methods Programs Biomed.

[27] Huang C-L, Dun J-F (2008). A distributed PSO–SVM hybrid system with feature selection and parameter optimization. Appl Soft Comput.

[28] Chuang L-Y, Yang C-H, Li J-C, Yang C-H (2011). A hybrid BPSO-CGA approach for gene selection and classification of microarray data. J Comput Biol.

[29] Lai C-M, Yeh W-C, Chang C-Y (2016). Gene selection using information gain and improved simplified swarm optimization. Neurocomputing.

[30] E. Pashaei, M. Ozen, N. Aydin. An application of black hole algorithm and decision tree for medical problem. In: 2015 IEEE 15th International Conference on Bioinformatics and Bioengineering (BIBE). 2015. p. 1–6.

[31] Sahu B, Mishra D (2012). A novel feature selection algorithm using particle swarm optimization for cancer microarray data. Procedia Eng.

[32] Xi M, Juan L, Liu L, Fan F, Wu X (2016). Cancer feature selection and classification using a binary quantum-behaved particle swarm optimization and support vector machine. Comput Math Methods Med.

[33] Tran B, Xue B, Zhang M (2018). A new representation in PSO for discretization-based feature selection. IEEE Trans Cybern.

[34] Chuang L-Y, Chang H-W, Tu C-J, Yang C-H (2008). Improved binary PSO for feature selection using gene expression data. Computat Biol Chem.

[35] Banka H, Dara S (2015). A Hamming distance based binary particle swarm optimization (HDBPSO) algorithm for high dimensionyal feature selection, classification and validation. Pattern Recognit Lett.

[36] Moradi P, Gholampour M (2016). A hybrid particle swarm optimization for feature subset selection by integrating a novel local search strategy. Appl Soft Comput.

[37] Jain I, Jain VK, Jain R (2018). Correlation feature selection based improved-Binary Particle Swarm Optimization for gene selection and cancer classification. Appl Soft Comput.

[38] Qasim OS, Algamal ZY (2018). Feature selection using particle swarm optimization-based logistic regression model. Chemom Intell Lab Syst.

[39] Li S, Wu X, Tan M (2008). Gene selection using hybrid particle swarm optimization and genetic algorithm. Soft Comput.

[40] Moteghaed NY, Maghooli K, Pirhadi S, Garshasbi M (2015). Biomarker discovery based on hybrid optimization algorithm and artificial neural networks on microarray data for cancer classification (in Eng). J Med Signals Sens.

[41] Moosa JM, Shakur R, Kaykobad M, Rahman MS (2016). Gene selection for cancer classification with the help of bees (in Eng). BMC Med Genom.

[42] Li B, Li J, Tang K, Yao X (2015). Many-objective evolutionary algorithms. ACM Comput Surv.

[43] Yu PL (1974). Cone convexity, cone extreme points, and nondominated solutions in decision problems with multiobjectives. J Optim Theory Appl.

[44] Theodoridis S, Koutroumbas K, Theodoridis S, Koutroumbas K (2009). Chapter 5—Feature selection. Pattern recognition.

[45] Blondel V, Guillaume J-L, Lambiotte R, Lefebvre E (2008). Fast unfolding of communities in large networks. J Stat Mech Theory Exp.

[46] De Meo P, Ferrara E, Fiumara G, Ricciardello A (2012). A novel measure of edge centrality in social networks. Knowl Based Syst.

[47] Qi X, Fuller E, Wu Q, Wu Y, Zhang C-Q (2012). Laplacian centrality: a new centrality measure for weighted networks. Inf Sci.

[48] Fernández-Martínez JL (2012). A brief historical review of Particle Swarm Optimization (PSO). J Bioinform Intell Control.

[49] M. Hall, E. Frank, G. Holmes, B. Pfahringer, P. Reutemann, I. Witten. The WEKA data mining software. http://www.cs.waikato.ac.nz/ml/weka.

[50] Moraglio A, Chio C, Togelius J, Poli R (2008). Geometric particle swarm optimization. J Artif Evol Applicat.

[51] Ghosh M, Begum S, Sarkar R, Chakraborty D, Maulik U (2019). Recursive Memetic Algorithm for gene selection in microarray data. Expert Syst Appl.

[52] Prasad Y, Biswas KK, Hanmandlu M (2018). A recursive PSO scheme for gene selection in microarray data. Appl Soft Comput.

[53] Chen K-H, Wang K-J, Wang K-M, Angelia M-A (2014). Applying particle swarm optimization-based decision tree classifier for cancer classification on gene expression data. Appl Soft Comput.

[54] Friedman M (1940). A comparison of alternative tests of significance for the problem of m rankings. Ann Math Stat.

[55] Nie NH, Hull CH, Jenkins JG, Steinbrenner K, Bent DH (1975). Statistical package for the social sciences.

[56] Liu H, Yu L (2005). Toward integrating feature selection algorithms for classification and clustering. IEEE Trans Knowl Data Eng.

[57] Chandrashekar G, Sahin F (2014). A survey on feature selection methods. Comput Electr Eng.

[58] De-Stefano FFC, Marrocco C, Scotto di Freca A (2014). A GA-based feature selection approach with an application to handwritten character recognition. Pattern Recognit Lett.

